# Are social isolation and loneliness associated with cognitive decline in ageing?

**DOI:** 10.3389/fnagi.2023.1075563

**Published:** 2023-02-23

**Authors:** Margalida Cardona, Pilar Andrés

**Affiliations:** ^1^Neuropsychology and Cognition Research Group, Department of Psychology, University of the Balearic Islands, Palma de Mallorca, Spain; ^2^Research Institute (IdISBa), Palma de Mallorca, Spain; ^3^Research Institute on Health Sciences (IUNICS), Palma de Mallorca, Spain

**Keywords:** social isolation, loneliness, cognition, ageing, cognitive decline, depression

## Abstract

**Objective:**

Social isolation and loneliness are associated with poor health (immunity, inflammation, etc.) in ageing. The purpose of this scoping review was to investigate the link between social isolation, loneliness (as distinct constructs, in contrast to previous published work) and cognition in cognitively healthy older adults.

**Method:**

We followed PRISMA-ScR guidelines. Our search, conducted between January 2017 and April 2021, yielded 2,673 articles, of which, twelve longitudinal studies were finally identified as meeting the inclusion criteria. Multiple cognitive functions (short-term and episodic memory, attention, and global cognitive functioning) were measured.

**Results:**

The results showed that both social isolation and loneliness were associated with poor cognition in ageing, with depression as a possible mediator between loneliness and poor cognition. Some studies also suggested that the link between social isolation, loneliness and cognitive decline may be bidirectional.

**Conclusion:**

We conclude that both social isolation and loneliness may have a different impact on cognition. While depression may be an important mediator between loneliness and cognitive decline, the lack of cognitive stimulation may be a greater mediator between social isolation and cognitive health.

## Introduction

Attachment theory suggests that human beings are born with a psychobiological system that motivates them to seek proximity to significant others in times of need (Bowlby, 1969, 1982). According to Bowlby, the goal of this attachment behavioral system, is to maintain adequate protection and support, which is accompanied by a sense of safety and security.

The health, life and genetic legacy of members of social species are threatened when they find themselves isolated (see Cacioppo and Hawkley, 2009 for a review). During the COVID-19 lockdown, for example, we experienced social isolation, and many people suffered the consequences of loneliness. During the pandemic, the dangers of the virus were prioritized. However, social isolation and loneliness can result in both short- and long-term health effects that cannot be ignored. Holt-Lunstad et al. (2015; see Leigh-Hunt et al., 2017 for a review), for instance, showed the association of social isolation and loneliness with a significantly increased risk of death from all causes. Likewise, being disconnected posed comparable danger to smoking 15 cigarettes a day, and was more predictive of early death than the effects of air pollution or physical inactivity. Holt-Lunstad et al. (2010) also showed that people who had strong social relationships had a 50% increased likelihood of survival than those with weaker ties. Similar effects have been observed using data from the Framingham Heart Study (Fowler and Christakis, 2008; Christakis and Fowler, 2009), whereby chances of becoming happy, depressed, or obese were mirrored by similar changes in the closest friend. When friends where considered, each person was asked to name one friend, and not all of these nominations were reciprocated. Results showed that the contagion phenomenon was especially strong if the friendship was mutual (both friends nominated each other as friend) (Fowler and Christakis, 2008).

The topic of social isolation and loneliness is considered so important that, prior to the pandemic, the UK appointed a Loneliness minister and published a national strategy for tackling loneliness in 2018 and the World Health Organization declared that loneliness is a major health concern worldwide.

In a recent review, Bzdok and Dunbar (2020) summarize the evidence showing that loneliness impairs the immune system, thus reducing resistance to disease and infections. For instance, Pressman et al. (2005) found that freshmen students who reported feeling lonely had a reduced immune system response when they were given a flu vaccine compared to students who felt socially well engaged. Moreover, those students with only four to 12 close friends had significantly poorer responses than those with more friends. Thus, feeling lonely and having few friends results in a particularly poor immune defense. Sarkar et al. (2012) also found that social bonds stimulate the release of the body’s natural killer cells, one of the white blood cells of the innate immune system whose core function is to destroy harmful bacteria and viruses. Finally, Cole and collaborators have shown that loneliness is associated with higher pro-inflammatory gene expression (Cole et al., 2007), indicating an upregulation of inflammatory signaling that can be a precursor for higher systemic inflammation (Irwin and Cole, 2011; Ligthart et al., 2018) and worse health (Slavich and Cole, 2013). To conclude, the more immersed in a community—with social connections—the happier, and healthier, people are. Friends tend to act as our social support and are condition *sine qua non* for health quality.

A second recent systematic review by Lam et al. (2021) has also shown abnormal brain structure (gray and white matter) and/or activity in the prefrontal cortex, insula, amygdala, hippocampus, and posterior superior temporal cortex associated with loneliness. Loneliness was also related to biological markers associated with Alzheimer’s disease pathology in two cross-sectional studies using PET imaging that found a significant relationship between loneliness and higher amyloid burden and greater tau pathology in right entorhinal cortex and right fusiform gyrus, especially in APOEε4 carriers (Donovan et al., 2016; d’Oleire Uquillas et al., 2018).

Both social isolation and loneliness refer to human connection and may have a relationship with cognition, but they are not synonymous. As stated by Palmer (2019), the term “social isolation” reflects an objective reality, meaning a factual deficit in a person’s social bonds and support. On the other hand, loneliness refers to a subjective feeling of discrepancy between one’s wishes of social contacts and actual interactions.

Research on social isolation over the years has shown that both objective (social isolation) and subjective (loneliness) components must be examined when investigating their association with health and wellbeing. They are two separate constructs that have shown only modest correlations (r ∼ 0.25–0.28; Palmer, 2019; also see de Jong-Gierveld and Havens, 2004; Cornwell and Waite, 2009; Coyle and Dugan, 2012) and may have independent negative effects on older adults’ mental health (Steptoe et al., 2013; Shankar et al., 2015; Cheung et al., 2021). Also, individuals may experience loneliness without also suffering social isolation, or vice versa (Valtorta and Hanratty, 2012). This difference has been identified as social asymmetry by McHugh Power and collaborators (McHugh Power et al., 2017, 2020).

Older adults’ health and wellbeing may be specially threatened by social isolation (Victor et al., 2002; Sundström et al., 2009). For example, they experience the loss of close others through ill health and bereavement, dislocation from their relatives due to increased familial mobility and greater difficulties engaging in social activity after retirement. Also, there is evidence that attachment style becomes more avoidant with age (Webster, 1997; Magai et al., 2001), especially when compared with younger adults (Diehl et al., 1998). Knowing that cognitive functioning constitutes a major outcome of older adults’ health and wellbeing, the aim of the current review was to explore further this link between social isolation and loneliness and cognitive functioning in healthy older adults.

Previous reviews have, however, addressed this question considering only separately the link between cognition and aspects of social relationships (Kuiper et al., 2016; Kelly et al., 2017; Evans et al., 2019) and between cognition and loneliness (Cacioppo and Hawkley, 2009; Boss et al., 2015).

There is however no published work considering both constructs as separate possible causes of cognitive decline. It was therefore important to investigate within the same study the specific contributions of social isolation and loneliness to cognition in older adults. The aim of the present review was to examine for the first time, in recent longitudinal studies (last 5 years), the relationship between social isolation, loneliness, as different concepts, and cognitive changes in later life.

## Methods

### Search strategy

To do this, we followed Preferred Reporting Items Systematic Reviews and Meta-Analysis for scoping reviews (PRISMA-ScR) guidelines (Tricco et al., 2018), with the pursue of achieving an appropriate organization and integrity of the work.

The question we wanted to investigate was whether there is a link between social isolation, loneliness and cognitive decline among older people. In other words, do isolated and/or lonely older adults present higher rates of cognitive decline than people with rich/active social life/connections? If such relationship does exist, could it be bidirectional?

### Inclusion criteria

To answer this question, the following inclusion criteria were established: studies (1) that examine the relationship between social isolation, loneliness and cognitive function, (2) using a longitudinal design, (3) including participants with a mean age ≥ 60, (4) with no diagnosis of cognitive impairment nor dementia, (5) reporting original data, (6) published in English in a peer-reviewed journal, (7) released between January 2017^1^ and April 2021. Reviews or opinion papers were excluded.

### Literature search strategy

Pubmed, Scopus, Web of Science (WOS), Medline and PsycInfo were the selected electronic databases through which the literature was searched. The search was conducted on April 2021, and it included the following terms: “social isolation,” “loneliness,” “ageing,” “older adult,” “elderly,” “cognition,” “cognitive function,” “cognitive processes,” “cognitive control,” “executive control,” “executive function,” in various combinations. For instance, in Scopus the following search was performed: (“social isolation”) AND (loneliness) AND (aging OR ageing OR “older adults” OR elderly) AND (cognition* OR “cognitive function*” OR “cognitive control” OR “cognitive processes*” OR “executive control” OR “executive function*”) AND (LIMIT-TO (PUBYEAR, 2021) OR LIMIT-TO (PUBYEAR, 2020) OR LIMIT-TO (PUBYEAR, 2019) OR LIMIT-TO (PUBYEAR, 2018) OR LIMIT-TO (PUBYEAR, 2017) AND (LIMIT-TO (LANGUAGE, “English”).

### Data collection and study selection process

**FIGURE 1:**
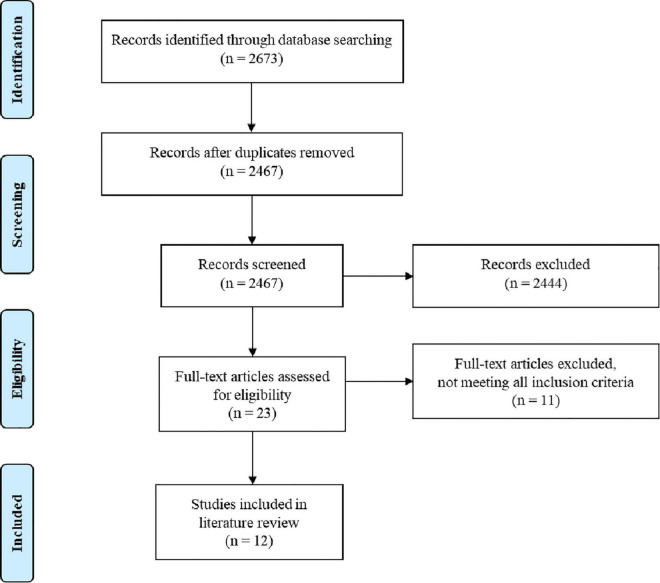
An overview of the study selection process.

As shown in Figure 1, the initial search yielded 2,673 results, from which 2,262 articles were identified in Scopus, 216 in PubMed, 116 in WOS, 63 in Medline and 31 in PsycINFO. A systematic filtering process was then conducted by the two authors, and in case of uncertainty, the final decision on inclusion was made through discussion.

First, 206 duplicates were removed. Titles and abstracts—and when in doubt also the methods—from the remaining 2,467 unique records were then screened, leaving 23 articles that were fully read and examined to determine eligibility for inclusion. Upon review, the final sample of this study is composed of 12 scientific publications (see Table 1). The 11 studies that were excluded for not fulfilling any of the criteria are listed in Table 2 along with the reason for exclusion.

**TABLE 1:** Relationship between social isolation and/or loneliness and cognitive function in older adults.

References	Objective, country and cohort	Characteristics of the sample	Loneliness/social isolation measurement	Cognitive measurement and function	Adjustments for covariates	Results
Evans et al. (2018)	To examine the relationship between social isolation and cognition in later life, and to consider the role of cognitive reserve in this relationship. Baseline and 2-year follow-up data Wales (UK). Cognitive Function and Ageing Study-Wales (CFAS-Wales)	*N* = 2224 Age ≥ 65 Mean (SD): 73.47 (±6.28) Sex: *N* (%) F: 1127 (50.67%) M: 1097 (49.33%)	Social isolation: Lubben Social Network Scale-6 (LSNS-6) Cognitive reserve: number of years in full time education, occupational complexity and cognitive activity	Cambridge Cognitive Examination (CAMCOG) Orientation, comprehension, expression, memory (remote, recent, and learning), attention and calculation, praxis, abstract thinking and perception	Age, gender, education (years), and physically limiting health conditions, such as sensory problems (hearing and eyesight), and ability to complete daily tasks alone	Being socially isolated in later life is associated with poor cognitive function, and cognitive reserve moderates this association at 2-year follow-up. Maintaining a socially active lifestyle in later life may enhance cognitive reserve and benefit cognitive function
Griffin et al. (2020)	To jointly examine isolation, loneliness, and cynical hostility as risk factors for cognitive decline in older adults. Follow-up every 2 years during 6 years USA Health and Retirement Study (HRS)	*N* = 6654 Age ≥ 65 Sex: *N* (%) F: 6026 (58.08%) M: 4350 (41.92%)	Social isolation: (1) frequency of contact with social network and (2) type of relationship Loneliness: Hughes Loneliness Scale Cynical hostility: Cook–Medley Hostility Inventory	Modified version of the Telephone Interview for Cognitive Status (TICS) Recall (i.e., immediate and delayed word recall) and mental status (i.e., the serial 7 s, backward counting from 20, and object, date, and president/vice president naming)	Age, education, sex, socioeconomic status (SES), and race), health status, and functional limitations	Loneliness and cynical hostility correlated with lower cognitive function, but none predicted change in cognitive function. Social isolation was associated with lower cognitive function and steeper decline in cognitive function Objective social isolation is a predictor of lower cognitive function and faster cognitive decline
Hajek et al. (2020)	To determine the link between perceived social isolation and cognitive functioning longitudinally Germany German Ageing Survey (DEAS)	*N* = 6420 Age ≥ 40 Mean (SD): 65.0 (±10.7) Sex: *N* (%) F: 3228 (50.3%) M: 3192 (49.7%)	Perceived social isolation: assessed using a scale by Bude and Lantermann	Digit symbol test, adapted from the digit symbol substitution Test Perceptual motor speed and processing speed of visual perception and information	Age, family status, household net equivalent income, labor force participation, self-rated health, physical functioning, physical illnesses, loneliness (De Jong Gierveld scale) and depressive symptoms (15-item version of the CES-D)	Increases in social isolation were associated with decreases in cognitive functioning longitudinally
Lara et al. (2019)	To examine the association of loneliness and social isolation on cognition over a 3-year follow-up period in middle- and older-aged adults Spain Collaborative Research on Ageing in Europe (COURAGE in Europe) Study	*N* = 1691 Age ≥ 50 Mean (SD): 64.5 (± 9.8)	Social isolation: measured considering: being married or cohabiting with a partner (or not); having less than monthly contact with children, other immediate family or friends; participating in any organizations, religious groups, or committees Loneliness: 3-item UCLA Loneliness Scale	Forward and backward digit span (Wechsler Adult Intelligence Scale), word list memory tasks (CERAD), animal naming task and a composite cognitive score Immediate recall, delayed recall, verbal fluency and episodic memory	Age, education, sex, level of physical activity, alcohol consumption, and disability. Additionally three chronic conditions were measured: depression in the previous 12 months, diagnosis of stroke and diabetes	Both loneliness and social isolation are associated with decreased cognitive function over a 3-year follow-up period The effect of loneliness and social isolation on cognition remained unchanged after the exclusion of individuals with depression, supporting the notion that loneliness and social isolation are not merely makers of depressive symptoms
Luchetti et al. (2020)	To test whether loneliness is associated with the risk of cognitive impairment up to 11 years later in a European sample of middle-aged and older adults. Austria, Belgium, Denmark, France, Germany, Greece, Italy, Spain, Sweden, Switzerland, Netherlands, and Israel. Survey of Health, Ageing and Retirement in Europe (SHARE)	*N* = 14114 Age ≥ 50 Mean (SD): 63.61 (±9.33) Sex: *N* (%) F: 7720 (54.7%) M: 6394 (45.3%)	Perceived loneliness: single-item measure of loneliness (CES-D) at baseline and three-item version of the UCLA loneliness scale at follow-ups from 2011	Immediate and delayed recall of 10 common words and naming as many animals as possible in 60 s. Memory recall task and fluency	Age, sex, educational level, social isolation, clinical and behavioral covariates, health-related activity limitations, and depression symptoms (using the EURO-D scale)	Feeling lonely increased the risk of developing cognitive impairment up to 11 years later The association remained significant in accounting for age, sex, education, clinical and behavioral risk factors, health-related activity limitations, social isolation and depressive symptoms
McHugh Power et al. (2019)	To investigate potential cross-lagged associations between sustained attention and loneliness, measured at baseline and again after 4 years Ireland Irish Longitudinal Study of Ageing (TILDA)	*N* = 6239 Age ≥ 50 Mean (SD): 63.05 (±9.22) Sex:% F: 54.6% M: 45.4%	Loneliness: specified as a latent factor with three indicators: the items constituting the 3-item version of the UCLA Loneliness Scale: “I feel left out,” “I feel isolated,” and “I lack companionship”	Sustained Attention to Response Task (SART) sustained attention	Age, sex, education (“no qualification,” “intermediate qualification,” and “degree qualification or higher”), depressive symptomatology (CES-D, with the item inquiring about loneliness removed)	While sustained attention at baseline predicted loneliness 4 years later, the converse, that loneliness would predict sustained attention, was not supported
McHugh Power et al. (2020)	To evaluate the relationship between loneliness and cognitive functioning, and whether depressive and anxiety symptoms have intermediate roles therein Ireland Irish Longitudinal Study of Ageing (TILDA). Data was collected at three time-points 2 years apart	*N* = 7433 Age ≥ 50 Mean (SD): 63.99 (±9.83) Sex: *N* (%) F: 3966 (53.36%) M: 3467 (46.64%)	Loneliness: 5-item version of the UCLA Loneliness Scale Depressive symptoms: 20-item CES-D scale Anxiety symptoms: Hospital Anxiety and Depression Scale Anxiety (HADS-A)	Measured as a latent factor, with four indicators: measures of immediate and delayed word recall (word list), verbal fluency (animal naming task) and a global measure Immediate and delayed word recall, verbal fluency, attention, orientation, memory, registration, calculation, language and praxis	Age, education level, sex and physical health (number of cardiovascular conditions, including angina, heart attack, heart failure, stroke, and abnormal heart rhythm etc.; and number of chronic conditions, including the above cardiovascular conditions, hypertension, diabetes, asthma, bronchitis, cancer, arthritis etc.)	Loneliness at time-point 1 predicted cognitive functioning at time-point 3, and anxiety symptoms at time-point 2. Depressive but not anxiety symptoms mediated the relationship between loneliness and cognitive functioning. However, the indirect effect of loneliness on cognitive functioning *via* depressive symptoms was small relative to the direct effect
Read et al. (2020)	To investigate associations between level and changes in social isolation and in memory in older men and women. Six measurement occasions every 2 years were conducted England (UK). English Longitudinal Study of Aging (ELSA)	*N* = 11233 Age ≥ 50 Mean (SD): 65.1 (±10.1) Sex: *N* (%) F: 6,123 (54.5%) M: 5,110 (45.5%)	Social isolation: index derived from five binary items	Word list recall in which the participant was asked to learn 10 common unrelated words Memory	Age, indicators of socioeconomic status (education, wealth, home ownership), and health-related behaviors (smoking, physical activity): all treated as time-invariant using values Limiting long-term illness, depressive symptoms, and whether working or doing voluntary work: all treated as time-varying covariates	Social isolation increased and memory decreased over time. The association between social isolation and memory decline is driven by the effect of social isolation on memory, rather than the reverse.
Wang et al. (2020)	To test the potential impact of loneliness amongst older old people on their cognitive function over a 20-year period UK Cambridge City over-75 s Cohort (CC75C) Study	*N* = 713 Age ≥ 75 Mean (SD): 86 (±4) Sex:% F: 71% M: 29%	Loneliness: single-item scale “Do you feel lonely?”; with response options “not at all lonely,” “slightly lonely,” “lonely” and “very lonely”	Mini-Mental State Examination (MMSE). Orientation, memory recall, working memory, attention, language, visual-spatial skills.	Age, sex, and education	Feeling slightly lonely and lonely were both associated with decline in cognitive function but neither of these associations were significant Loneliness was not a risk factor for cognitive function decline over a 20-year period. Loneliness did not exert long-term harmful effects on cognitive function in the oldest old
Yin et al. (2019)	To examine whether there is a bidirectional relationship between loneliness and cognitive function over a 10-year follow-up England (UK). English Longitudinal Study of Aging (ELSA)	*N* = 5885 Age ≥ 50 Mean (SD): 65.3 (±9.0) Sex: N (%) F: 3401 (55.4%) M: 2734 (44.6%)	Loneliness: abridged version of the revised UCLA Loneliness Scale	Word recall and verbal fluency tests Memory and verbal fluency	Age and sex, educational level, wealth, illness or disability that impaired their everyday life over an extended period Depression (using a combined algorithm of physician diagnosis and a positive score on the seven items of the CES-D scale, after excluding the loneliness item from the standard eight-item CES-D)	Higher loneliness is associated with poorer cognitive function at baseline and contributes to a worsening in memory and verbal fluency over a decade. These factors seem, however, to be partially intertwined, since baseline memory and its rate of decline also contribute to an increase in loneliness over time
Yu et al. (2020)	To examine the relationships of social isolation and loneliness on cognitive function among Chinese older adults over a 4-year follow-up period China China Health and Retirement Longitudinal Study (CHARLS)	*N* = 7761 Age ≥ 50 Mean (SD): 60.97 (±7.31) Sex:% F: 49.2% M: 50.8%	Social isolation: three items were combined to create an index of social isolation: married/not married; weekly contact with children; and participating in any social activities over the last month Loneliness: one single item included in the CES-D: “In the last week, how often did you feel lonely?”	Telephone Interview for Cognitive Status (TICS) and immediate word recall followed by delayed recall Mental status (orientation, visuospatial ability and numeric ability) and episodic memory	Demographic variables and behavioral, psychological, and clinical risk factors, age, gender, education, and area of residence (urban/rural). Depressive symptoms (measured with CES-D-9 (a modified CES-D-10 excluding the loneliness item). Chronic diseases (including hypertension, diabetes, and heart diseases), health behaviors/habits (including drinking and smoking), and disabilities (functional limitations in activities of daily living (ADL)	Social isolation was significantly associated with decreases in all cognitive function measures at follow-up even after controlling for loneliness and all confounding variables. Loneliness was significantly associated with cognitive decline at follow-up in the partially adjusted models. However, these associations became insignificant after additional confounding variables (chronic diseases, health behaviors, disabilities and depressive symptoms) were taken into account
Zhou et al. (2019)	To investigate the association between loneliness and cognitive impairment among older men and women in China over a 3-year follow-up period China Chinese Longitudinal Healthy Longevity Survey (CLHLS)	*N* = 6898 Age ≥ 65	Loneliness: one single question: “Do you feel lonely?” (item extracted from the CES-D)	Self or proxy-report at follow-up using a culturally adapted, Chinese version of the MMSE Orientation, reaction, calculation ability, recall, and language ability	Social-demographic variables, lifestyles, health status and social isolation: Age, education level, employment status, and body mass index (BMI), current smoking and current drinking, physical exercise. Health status, such as CVD, diabetes and activities of daily living (ADL) disability, both instrumental and basic ADL. Social isolation: assessed using the following three separate items: Living alone (yes or no), being married (yes or no) and having social support	Although elderly women more frequently reported feelings of loneliness, the impact of loneliness on cognitive impairment was significant among elderly men but not elderly women

**TABLE 2:** Excluded studies with reason for exclusion.

Excluded studies	
References	Reason for exclusion
Beller, J., and Wagner, A. (2018). Disentangling loneliness: differential effects of subjective loneliness, network quality, network size, and living alone on physical, mental, and cognitive health. *Journal of aging and health*, 30 (4), 521–539. https://doi.org/10.1177/0898264316685843	<50 and variables not relevant
Donovan, N. J., Qiong, W., Rentz, D. M., Sperling, R. A., Marshall, G. A., Glymour, M. M. (2017). Loneliness, depression and cognitive function in older U.S. adults. *International Journal of Geriatric Psychiatry*, 32 (5), 564–573. https://doi.org/10.1002/gps.4495	Inappropriate sample
Evans, I., Llewellyn, D. J., Matthews, F. E., Woods, R. T., Brayne, C., Clare, L., and CFAS-Wales research team (2019). Living alone and cognitive function in later life. *Archives of Gerontology and Geriatrics*, 81, 222–233. https://doi.org/10.1016/j.archger.2018.12.014	Variables not relevant (some cognitive impairment)
Fung, A., Lee, A., Cheng, S. T., and Lam, L. (2019). Loneliness interacts with family relationship in relation to cognitive function in Chinese older adults. *International psychogeriatrics*, 31 (4), 467–475. https://doi.org/10.1017/S1041610218001333	Cross-sectional
Jang, Y., Choi, E. Y., Park N. S., Chiriboga, D. A., Duan, L. and Kim, M. T. (2021). Cognitive health risks posed by social isolation and loneliness in older Korean Americans. *BMC Geriatrics*, 21, 123. https://doi.org/10.1186/s12877-021-02066-4	Cross-sectional
Lam, C. L. M., Junhong, Y. and Lee, T. M. C. (2017). Perceived loneliness and general cognitive status in community-dwelling older adults: the moderating influence of depression. *Aging, Neuropsychology, and Cognition* 24 (5), 471–480. https://doi.org/10.1080/13825585.2016.1226246	Cross-sectional
McHugh Power, J. E., Kenny, R. A., Lawlor, B. A., Steptoe, A., and Kee, F. (2017). The discrepancy between social isolation and loneliness as a clinically meaningful metric: findings from the Irish and English longitudinal studies of ageing (TILDA and ELSA). *International journal of geriatric psychiatry*, 32 (6), 664–674. https://doi.org/10.1002/gps.4509	Cross-sectional and variables not relevant
McHugh Power, J. E., Sjöberg, L., Kee, F., Kenny, R. A., and Lawlor, B. (2019b). Comparisons of the discrepancy between loneliness and social isolation across Ireland and Sweden: findings from TILDA and SNAC-K. *Social psychiatry and psychiatric epidemiology*, 54 (9), 1079–1088. https://doi.org/10.1007/s00127-019-01679-w	Cross-sectional and variables not relevant
Oremus, M., Tyas, S. L., Maxwell, C. J., Konnert, C., O’Connell, M. E., and Law, J. (2020). Social support availability is positively associated with memory in persons aged 45–85 years: A cross-sectional analysis of the Canadian Longitudinal Study on Aging. *Archives of Gerontology and Geriatrics*, 86, 103962. https://doi.org/10.1016/j.archger.2019.103962	Cross-sectional
Sin, E., Shao, R., and Lee, T. (2021). The executive control correlate of loneliness in healthy older people. *Aging and Mental Health*, 25 (7), 1224–1231. https://doi.org/10.1080/13607863.2020.1749832	Cross-sectional
Yang, R., Wang, H., Edelman, L. S., Tracy, E. L., Demiris, G., Sward, K. A., and Donaldson, G. W. (2020). Loneliness as a mediator of the impact of social isolation on cognitive functioning of Chinese older adults. *Age and ageing*, 49 (4), 599–604. https://doi.org/10.1093/ageing/afaa020	Cross-sectional

#### Extracting and charting the data

The following information from each of the included studies was extracted (see Table 1): authors and year of publication, country and setting in which it was carried out, objective of the investigation, study design, characteristics of the sample, loneliness and/or social isolation measurement, cognitive domains measurement, covariates, and results obtained.

## Results

### Study characteristics

#### Design

Only longitudinal studies were included in this scoping review, as they represent the best way to investigate associations between risk factors and cognitive decline and try to establish directional relationships (e.g., Boss et al., 2015). Follow-up periods for all 12 longitudinal studies ranged from 3 (Zhou et al., 2019; Hajek et al., 2020) to 11 years (Luchetti et al., 2020). All studies reported findings from participants enrolled in large, population-based studies, being these the Survey of Health, Ageing and Retirement in Europe (SHARE; Luchetti et al., 2020), the English Longitudinal Study of Ageing (ELSA; Yin et al., 2019; Read et al., 2020), the Cognitive Function and Ageing Study-Wales (CFAS-Wales; Evans et al., 2018), the Cambridge City over-75s Cohort Study (CC75C; Wang et al., 2020), the Irish Longitudinal Study of Ageing (TILDA; McHugh Power et al., 2019, 2020), the Collaborative Research on Ageing in Europe study in Europe (COURAGE; Lara et al., 2019), the Health and Retirement Study (HRS; Griffin et al., 2020), the Chinese Longitudinal Healthy Longevity Survey (CLHLS; Zhou et al., 2019), the China Health and Retirement Longitudinal Study (CHARLS; Yu et al., 2020) and the German Ageing Survey (DEAS; Hajek et al., 2020).

#### Sample and location

Eight studies were conducted in Europe, including Germany (Hajek et al., 2020), Spain (Lara et al., 2019), Ireland (McHugh Power et al., 2019, 2020), and the UK (Evans et al., 2018; Yin et al., 2019; Read et al., 2020; Wang et al., 2020). Two studies were carried out in China (Zhou et al., 2019; Yu et al., 2020) and one in the USA (Griffin et al., 2020). Lastly, Luchetti et al.’s (2020) sample was formed using data from several countries—Austria, Belgium, Denmark, France, Germany, Greece, Italy, Spain, Sweden, Switzerland, Netherlands, and Israel.

In all studies samples included both genders, and the number of participants per study ranged from 713 (Wang et al., 2020) to 14,114 (Luchetti et al., 2020) for a total of 77,265 participants across all studies. All studies’ participants were independently living older adults. Most participants were described as generally healthy, although some suffered from chronic physical diseases such as hypertension, diabetes, cardiovascular conditions, arthritis as well as psychological disorders—such as depression and anxiety.

#### Measurement of loneliness

From the nine studies investigating loneliness, some utilized the UCLA loneliness scale (Russell, 1996; McHugh Power et al., 2020) or a three-item shortened version of it (the Hughes loneliness scale, Hughes et al., 2004; Lara et al., 2019; Griffin et al., 2020; Luchetti et al., 2020; second wave; McHugh Power et al., 2019; Yin et al., 2019). This Likert style questionnaire was designed to measure general feelings of dissatisfaction with one’s social interactions, and it includes questions such as “How often do you experience loneliness?” The other researchers used single questions. Zhou et al. (2019) and Wang et al. (2020) used yes/no format questions such as “do you feel lonely?.” Luchetti et al.’s (2020) single—item question was part of the abbreviated version of the Center for Epidemiological Studies Depression scale (CESD; Prince et al., 1999). Participants were asked: “How often have you experienced the following feelings over the last week: I felt lonely?.” Response options were: 1 = Almost all of the time; 2 = Most of the time; 3 = Some of the time; 4 = Almost none of the time. Yu et al. (2020) also used this single question from the CESD.

#### Measurement of social isolation

Six studies investigated social isolation. From these, Hajek et al. (2020) assessed perceived social isolation using the Bude and Lantermann’s (2006) scale, which includes 4 items. Each item ranges from 1 (strongly agree) to 4 (strongly disagree). Some other authors used indexes. For example, Lara et al. (2019) and Read et al. (2020) used an index based on the one proposed by Shankar et al. (2011), including 5 binary questions such as “did the responded live alone?,” with scores ranging from 0 to 5, and higher scores representing greater social isolation. Yu et al. (2020) combined three items to create an index of social isolation, adapted from previous research (Glei et al., 2012; Steptoe et al., 2013). One point was assigned, for example, if participants were not married or had less than weekly contact (by phone, in person, or by e-mail) with children. Scores of social isolation ranged from 0 to 3, with higher scores indicating greater isolation. Finally, Evans et al. (2018) used the Lubben Social Network scale-6 (LSNS-6, Lubben et al., 2006), a self-report standardized measure of social engagement including family and friends, constructed of three sets of questions. The three items assess the number of relatives/friends the participant sees or hears from at least once a month, could call on for help, and can speak with about private matters. Responses are collected using a six category response, in which the participant indicates the number of relatives/friends available. Response scores range from 0 (no relatives/friends) to 5 (nine or more relatives/friends). The overall scores for each six questions are summed and range from 0 to 30, with higher scores indicating lower social isolation. A score of ≤ 12 may be taken to indicate the presence of social isolation. Griffin et al. (2020) used a scale to measure frequency of three types of contact with social network (Smith et al., 2012, 2020). Participants had to rate how often they (a) met up (include both arranged and chance meetings), (b) spoke on the phone, (c) wrote to or emailed their children, other family members, and friends, respectively. Options included three or more times a week, once or twice a week, once or twice a month, every few months, once or twice a year, less than once a year, or never (Smith et al., 2013).

#### Measurement of cognitive function

Episodic memory was assessed in different studies *via* word recall (Lara et al., 2019; Luchetti et al., 2020; McHugh Power et al., 2020; Read et al., 2020; Yu et al., 2020). For example, Lara et al. (2019); Luchetti et al. (2020), and Read et al. (2020) used the 10-word list immediate and delayed verbal recall from the CERAD (Morris et al., 1989). In terms of attention, Hajek et al. (2020) used the digit symbol test adapted from the digit symbol substitution test (Wechsler, 1955). McHugh Power et al. (2019) used the Sustained Attention to Response Task (SART, Robertson et al., 1997), where a series of single digits between 1 and 9 are sequentially presented and participants are instructed to press a keyboard key as soon as possible (with response time noted) for each digit presented, except if the digit is 3. Lara et al. (2019) used the digit span from the Wechsler Adult Intelligence Scale (Wechsler, 1955). Verbal fluency was also assessed by Lara et al. (2019); Yin et al. (2019), Luchetti et al. (2020), and McHugh Power et al. (2020). Other studies assessed global cognitive function using the Mini Mental State Examination (MMSE, McHugh Power et al., 2020; Wang et al., 2020; Zhou et al., 2019). It is noteworthy that this test has been criticized and is being replaced by the Montréal Cognitive Assessment (MoCA) as it is more sensitive and specific to detect early cognitive impairment than the MMSE (Damian et al., 2011; Ciesielska et al., 2016; Pinto et al., 2019; Jia et al., 2021). Evans et al. (2018) used the Cambridge Cognitive Examination (CAMCOG), a standardized instrument used to measure orientation, language, memory, praxis, attention, abstract thinking, perception, and calculation (Roth et al., 1986). Finally, Griffin et al. (2020) used the Modified version of the telephone interview for cognitive status (TICS; de Jager et al., 2003), which includes questions of orientation, repetition, naming, and calculations.

#### Social isolation and cognition

Six studies from our sample examined the relationship between social isolation and cognitive functioning. Hajek et al. (2020), after examining a German sample of 6,420 people, concluded that increases in social isolation were associated with decreases in cognitive function, specifically perceptual motor speed and processing speed. Likewise, Read et al. (2020) revealed a link between social isolation and memory decline in 11,233 later-life adults in England, suggesting that the former seems to affect the latter, but not the reverse. The study by Evans et al. (2018) explored this relationship in 2,224 Welsh older adults, assessing multiple domains and showed that being socially isolated might lead to poor cognitive functioning. This research also analyzed the role that cognitive reserve could play in the relationship between social isolation and cognition in later life, showing that, although no link was found when explored cross-sectionally, at 2-year follow up, cognitive reserve seemed to moderate the association. Particularly, the association between social isolation and cognitive change was non-significant for those participants whose former main employment was considered complex (i.e., doctor or lawyer) and those whose social and economic class were higher.

Griffin et al. (2020) examined data about objective social isolation, loneliness and recall from 6654 US individuals during 6 years with follow-ups every 2 years. Concerning social isolation, their results suggest that it is a predictor of lower cognitive functioning and it accelerates cognitive decline. In the same way, after analyzing data from 7,761 Chinese adults over 50 years, Yu et al. (2020) concluded that social isolation was significantly linked to cognitive decline in all cognition measures (i.e., episodic memory, orientation and visuospatial and numeric ability) at follow-up, even after taking into account loneliness and other covariates such as chronic diseases or depression. Lara et al. (2019) evaluated 1,691 Spanish participants aged 50 or older to determine whether loneliness and/or social isolation were related to changes in cognition over a 3-year follow up period. They found that individuals with a higher isolation index tended to present lower scores in verbal fluency, forward digit span and in the composite cognitive score.

#### Loneliness and cognition

Nine studies from our sample investigated the association between loneliness and cognitive function. Some (Lara et al., 2019; McHugh Power et al., 2019; Luchetti et al., 2020) reported that loneliness might be a predictor of cognitive changes over time, while others (Griffin et al., 2020; Wang et al., 2020; Yu et al., 2020) did not. The study by Yin et al. (2019) suggests a bidirectional relationship between both domains and McHugh Power et al. (2019) found that attention may affect loneliness and not reversely. Finally, Zhou et al. (2019) concluded that the link between loneliness and cognitive decline over time was significant among men but not women.

Lara et al.’s (2019) study concluded that loneliness had a significant association with lower scores in immediate and delayed recall, verbal fluency, backward digit span and the composite cognitive score that evaluated overall cognition. The research by Luchetti et al. (2020) is the study with the largest sample (14,114 individuals assessed over 11 years) and culturally the most heterogeneous (comprising 12 European countries). It evaluated memory recall and verbal fluency, and the results suggest that feeling lonely increases the risk of suffering cognitive impairment regardless of all covariates controlled (i.e., social isolation, depressive symptoms).

Moreover, Yu et al. (2020) found that loneliness had a significant association with a decline in episodic memory, orientation and visuospatial and numeric ability before controlling for variables such as chronic diseases, health behaviors, disabilities, and depression. However, after controlling for them, this relationship became insignificant. In the same vein, Griffin et al. (2020) reported that, unlike social isolation, loneliness does not seem to predict changes in cognition longitudinally, although it correlated with lower cognitive function cross-sectionally. Wang et al.’s (2020) study is the only one whose sample is composed solely by individuals aged 75 or older evaluated over a 20-year period, reason why its sample (713) is the smallest. The results showed that loneliness did not seem to be a significant long-term harmful risk factor for cognitive decline among the older old.

Finally, Zhou et al. (2019) focused their efforts on analyzing whether the association between loneliness and cognitive function varied among 6,898 Chinese men and women 65 years and over. Notwithstanding older women reported feelings of loneliness more frequently, the repercussion of loneliness on cognitive decline over time was significant among older men but not women.

#### Is the relationship bidirectional?

A remaining question from previous reviews was whether the association between social isolation and cognition or between loneliness and cognition might be bidirectional. The fact that the reviewed studies in our scoping review were longitudinal may contribute to answer this question.

When analyzing the association between loneliness and cognitive function (memory and verbal fluency), Yin et al. (2019) observed that higher loneliness predicted poorer memory and verbal fluency at baseline and influenced such cognitive domains in a negative way after a 10-year follow up. Besides, worse baseline memory (but not verbal fluency) and its rate of decline over time seemed to contribute to an intensification of loneliness at follow-up. Additionally, higher baseline memory seemed to predict a slower change in loneliness, revealing a bidirectional relationship among loneliness and memory. In another study, McHugh Power et al. (2020) found that cognitive functioning at wave 1 predicted loneliness at wave 3 and loneliness at wave 1 predicted cognitive functioning at wave 3. Finally, McHugh Power et al. (2019) also hypothesized a bidirectional relationship between loneliness and sustained attention. Nevertheless, sustained attention at baseline predicted loneliness at 4-year follow-up, but reverse results, loneliness predicting sustained attention, were not found. This study is unique in that it is the only study in which solely cognitive function prognosticates loneliness longitudinally.

#### The role of depressive symptoms

Five out of the 12 reviewed studies considered the role of depression as a possible mediator between social isolation, loneliness and cognitive decline. In the study by Lara et al. (2019) depression in the previous 12 months was assessed with an adapted version of the CIDI 3.0 (Haro et al., 2006). Lara et al. (2019) concluded that the results were not affected by depression, as they remained unchanged after excluding individuals with depression. Similarly, Yin et al. (2019) reported that, albeit loneliness and depression measured using seven items of the CES-D (Radloff, 1977), seemed to be intimately linked, ultimately, they were independent and loneliness might be associated with memory decline over a decade by itself, despite of depressive symptoms.

Three studies showed however a significant link between loneliness and depression (McHugh Power et al., 2019, 2020; Yu et al., 2020). The first showed that higher levels of depressive symptomatology (measured using the validated and reliable 20-item CES-D scale, Radloff, 1977) predicted higher levels of loneliness and worse performance on sustained attention. McHugh Power et al. (2020) also observed that depressive (20-item CES-D scale), but not anxiety, symptoms mediated the relationship between loneliness and cognitive functioning. Finally, Yu et al. (2020) demonstrated that the association between loneliness (not social isolation) and cognitive decline became insignificant after depressive symptoms (measured with the 10-item CESD-10, Radloff, 1977) were taken into account.

## Discussion

The aim of this scoping review was to investigate for the first time possible associations between social isolation, loneliness, as separate constructs, and cognitive function in cognitively healthy older adults. To do this, we reviewed longitudinal studies investigating social isolation, loneliness and multiple cognitive functions. Studies were carried out in three continents (Europe, Asia, and America) and included large samples with a total of 77,265 participants across studies.

Results from the selected studies revealed that social isolation, loneliness and cognition are related. The relationship between social isolation, understood as an *objective* manifestation of lack of social bonds and support, and cognition seems robust, with results congruently (6 out of 6 studies) showing that it is negatively associated with cognitive functions in older populations. Previous reviews assessing studies published before 2017 had shown some heterogeneity in the results, depending on variations in approaches to measuring social activity and social networks across studies (Kuiper et al., 2016; Kelly et al., 2017; Evans et al., 2019). Our scoping review on recent longitudinal studies however adds consistent evidence to the idea of a protective effect of social connection through life on cognitive functions.

The results of the recent studies looking at the effect of loneliness on cognitive function seem to be less consistent. Despite the common knowledge that “loneliness kills,” the relationship between loneliness, understood as the *subjective* feeling of discrepancy between one’s wishes of social contacts and actual interactions and cognition is only clearly shown in 4 out of 9 studies. While some studies identify loneliness as a predictor of cognitive function (Lara et al., 2019; Yin et al., 2019; Luchetti et al., 2020; McHugh Power et al., 2020), others do not (McHugh Power et al., 2019; Griffin et al., 2020; Wang et al., 2020; Yu et al., 2020) or show it only for men (Zhou et al., 2019). Moreover, this relationship may be, at least partially, mediated by depression (McHugh Power et al., 2019, 2020; Yu et al., 2020).

Measuring loneliness is not a simple issue. It was measured by the UCLA loneliness scale in one study (McHugh Power et al., 2020) and by a three-item shortened version of it in five studies (Lara et al., 2019; Griffin et al., 2020; Luchetti et al., 2020; second wave; McHugh Power et al., 2019; Yin et al., 2019). The other researchers used single questions (Zhou et al., 2019; Luchetti et al., 2020; Wang et al., 2020). As an example of how prevalent this approach is, from the 10 studies examining loneliness in the ageing population in Boss et al.’s (2015) meta-analysis, seven of those made use of single-item questions to measure loneliness while the remaining three used either the 3-item, short form of the Revised-UCLA Loneliness Scale (Shankar et al., 2013) or the six-item De Jong-Gierveld Loneliness Scale (Wilson et al., 2007; Schnittger et al., 2012). Also, recent research has shown that the subcomponents of loneliness scales were notably decoupled by the confinement during COVID, supporting the notion that loneliness is not a unitary, isolated construct but rather represents a cluster of different experiences of social integration and socioemotional states (Bartrés-Faz et al., 2021). It is then possible that to capture feelings of loneliness and detect associations with cognitive decline, a full scale should be used (as was the case only in the study by McHugh Power et al., 2020).

Cacioppo and Patrick (2008) define loneliness as a signal that one’s connections to others are weakening and to motivate the repair and maintenance of connections to others that are needed for our health, wellbeing and survival of our genes. This definition considers that loneliness evolved to improve survivability when socially isolated, through hypervigilance and increasing motivation to connect with others (also see Hawkley and Capitanio, 2015). Putting together the results from the current review and previous studies, the findings of depression as a mediator between loneliness and cognitive decline found in some studies (McHugh Power et al., 2019, 2020; Yu et al., 2020) would be compatible with this conception. Importantly, loneliness has been previously found to be closely associated with depressive symptoms (Cacioppo et al., 2006, 2010; Sjöberg et al., 2013). The link between depression and loneliness is clearly established by authors that conceive depression as an evolutionarily conserved mechanism to terminate separation distress (Watt and Panksepp, 2009; Panksepp and Watt, 2011; Watt, 2014; also see Slavich, 2020; social safety theory), placing social loss at the center of the neurobiological dynamics (e.g., inflammation, altered HPA axis functioning, declining neurotrophins prosocial peptides and amines) driving depression. Also, two studies excluded from this scoping review (see Table 2) demonstrated the key role played by depression as a mediator between loneliness and cognition. Donovan et al.’s (2017) study with 8,382 USA participants aged 65 or older concluded that, after 12 years, the effect of loneliness on cognitive function became marginally significant after controlling for depression, and that the latter is associated with a more rapid cognitive decline. Similarly, Lam et al. (2017) revealed that, only among the individuals who reported higher levels of depressive symptoms, loneliness was associated with poorer cognitive function.

Interestingly, this link with depressive symptoms was not observed for social isolation. In the scoping review by Courtin and Knapp (2017) on social isolation, loneliness and health in old age, 25 studies looked at the link between loneliness and depression and 3 looked at the link between social isolation and depression. The evidence reviewed clearly showed that loneliness is a strong risk factor for depression in old age, even after controlling for a number of covariates such as demographic characteristics, marital status, social isolation and psychosocial risk factors. The detrimental effect of living alone on depression was more often due to loneliness for men than for women. The evidence for a link between social isolation and depression was however weaker. This evidence suggests that the subjective experience of loneliness may be more strongly related to depression than the objective isolation *per se*, and that the link between social isolation and cognitive dysfunction is less mediated by depression.

Considering the studies that investigated social isolation and loneliness simultaneously, Griffin et al.’s (2020) and Yu et al.’s (2020) results also suggest that social isolation may have a more substantial impact than loneliness on cognitive decline. For instance, correlations between loneliness and cognitive function were non-significant after controlling for a wide range of demographic and psychosocial risk factors thought to influence loneliness. Consistent with our findings, recent research has suggested that social isolation is more associated with objective cognitive impairment outcomes, while loneliness is more associated with a subjective dimension of cognitive function (Jang et al., 2021; see Table 2). This is also consistent with Boss et al.’s (2015) review showing that, when social and emotional loneliness were examined, the first seemed to have a stronger correlation with global cognitive function.

These findings suggest that both social isolation and loneliness may impact cognitive health but probably in a different way, with a stronger weight on cognitive reserve for the first and on emotional wellbeing for the second. A possible explanation of the link between social isolation and cognitive decline would have to do with the “use it or lose it” perspective (Hultsch et al., 1999; Salthouse, 2006). Park and Bischof (2013) reviewed the evidence suggesting that engagement in an environment that requires cognitive effort may facilitate cognitive function in older adults. This view, linked to the notion of neuroplasticity (also see Greenwood and Parasuraman, 2010) suggests that the brain can be conceived as a muscle and that engagement in intellectual, social and physical activities stimulates the brain. If engagement in everyday activities is absent, it may result in disuse of the brain which will result in decline of cognitive functions. The seemingly stronger association between cognition and social isolation than between loneliness and cognition would go in favor of this explanation.

On the other hand, although the empirical literature regarding the underlying biological mechanisms involved in social isolation and loneliness is scarce and not fully consistent (Palmer, 2019), there is some evidence suggesting that, biologically, loneliness may trigger immune system impairment, chronic inflammation, hypertension, hyperlipidemia (Cunningham, 2013; Cacioppo et al., 2014, 2015; Hawkley and Capitanio, 2015), hypercortisolism (Boss et al., 2015) and prolonged activation of the hypothalamic–pituitary–adrenal (HPA) axis, which in turn could lead to a decrease in dendritic arborization in the prefrontal cortex (CPF) and hippocampus (Cacioppo and Hawkley, 2009). Nonetheless, many of these neurological and physiological changes are linked to ageing itself, and, hence, the mixed effects of loneliness, social isolation and ageing may be even more complex.

As for the direction of the relationship, the idea of this review came from the interest in the consequences of the isolation experienced during the COVID-19 pandemic, with the question of whether social isolation might be detrimental to cognition. In that sense, the direction of the effect may be conceived from social isolation and loneliness to cognition. However, there is evidence suggesting that the effect can also go in the opposite direction, i.e., poor cognition can lead to isolation and loneliness, known as reverse causality. It has been suggested that older adults who experience declining self-efficacy and loss of attachment relationships defensively place more emphasis on independence and self-reliance and less on interdependence (Zhang and Labouvie-Vief, 2004). Yin et al. (2019) reached such a conclusion following their result of a higher rate of memory decline predicting loneliness at follow-up and vice versa. Possible explanations for this is that poor cognition may generate feelings of insecurity. Schnittger et al. (2012), for example, showed that decreased verbal fluency was a significant predictor of social loneliness. Poor communication skills may discourage conversation, hinder meaningful relationships, and thus increase loneliness. Another recent study (Sin et al., 2021; see Table 2), also found that a dysfunction in working memory and planning might prognosticate higher perceived loneliness. McHugh Power et al. (2019) also revealed this reverse association, demonstrating that sustained attention at baseline predicted loneliness 4 years later.

Previous reviews had mentioned the difficulty to solve this question due to the great proportion of cross sectional studies (see for example, Boss et al., 2015; Courtin and Knapp, 2017). Our review minimized this risk by excluding participants with cognitive impairment of dementia and by excluding cross-sectional studies. To conclude, it is likely that the relationship between social isolation loneliness and cognitive decline may be bidirectional.

As limitations of the present study, the heterogeneity observed in measures of social isolation, loneliness and cognitive functions in the reviewed studies makes it difficult to carry out more specific analyses (e.g., meta-analysis). Also, only 12 studies fulfilled the inclusion criteria. As strengths, only powerful longitudinal studies evaluating cognitively healthy older participants were reviewed. This is the best method to explore causal relationships between the studied variables. Moreover, studies from three different continents were included, which helps generalizing the results. Finally, a key strength is that we considered both social isolation and loneliness as measures of objective and subjective isolation, respectively, which should contribute to a better understanding of differential effects of both constructs on cognitive decline in ageing.

## Conclusion

After considering the 12 longitudinal studies that comprise large heterogeneous, and culturally diverse populations, we conclude that both loneliness and social isolation, common among older adults, may be associated with cognitive decline. It seems that the relationship between social isolation and cognitive decline may be stronger than the link between loneliness and cognition, possibly mediated by depression.

We also notice that both constructs are complex, with a diversity of definitions and measurements, and require more nuanced examination, with special attention to the specific nature or forms of social isolation, loneliness and their interactions in affecting mental health and cognition. Additional research is necessary to determine more precisely the causality and biological mechanisms implied in the association between social isolation, loneliness and cognitive functioning.

## Data availability statement

The original contributions presented in this study are included in the article/supplementary material, further inquiries can be directed to the corresponding author.

## Author contributions

Both authors listed have made a substantial, direct, and intellectual contribution to the work, and approved it for publication.

## Footnotes

1 The final time period within the bibliographical searches in previous reviews was January 2017 [in Kelly et al.’s (2017) study].
